# Genetic diversity and molecular epidemiology of human rhinoviruses in South Africa

**DOI:** 10.1111/irv.12264

**Published:** 2014-07-03

**Authors:** Marthi A Pretorius, Stefano Tempia, Florette K Treurnicht, Sibongile Walaza, Adam L Cohen, Jocelyn Moyes, Orienka Hellferscee, Ebrahim Variava, Halima Dawood, Meera Chhagan, Sumayya Haffjee, Shabir A Madhi, Cheryl Cohen, Marietjie Venter

**Affiliations:** a Centre for Respiratory Diseases and Meningitis, National Institute for Communicable Diseases of the National Health Laboratory ServiceJohannesburg, South Africa; b Department of Medical Virology, University of PretoriaPretoria, South Africa; c Influenza Division, Centers for Disease Control and PreventionAtlanta, GA, USA; d Influenza Division, Centers for Disease Control and PreventionPretoria, South Africa; e Influenza Programme, Centers for Disease Control and Prevention–South AfricaPretoria, South Africa; f Faculty of Health Sciences, School of Public Health, University of the WitwatersrandJohannesburg, South Africa; g Department of Medicine, Klerksdorp Tshepong HospitalKlerksdorp, South Africa; h Department of Medicine, Faculty of Health Sciences, University of the WitwatersrandJohannesburg, South Africa; i Department of Medicine, Pietermaritzburg Metropolitan HospitalPietermaritzburg, South Africa; j Department of Medicine, University of KwaZulu NatalPietermaritzburg, South Africa; k Department of Paediatrics, University of KwaZulu NatalPietermaritzburg, South Africa; l School of Pathology, University of KwaZulu NatalPietermaritzburg, South Africa; m Medical Research Council, Respiratory and Meningeal Pathogens Research Unit, Faculty of Health Sciences, University of the WitwatersrandJohannesburg, South Africa; n Department of Science and Technology/National Research Foundation, Vaccine Preventable Diseases, University of the WitwatersrandJohannesburg, South Africa; o Global Disease Detection, Centres for Disease Control and PreventionPretoria, South Africa

**Keywords:** Disease association, genetic diversity, rhinovirus, South Africa

## Abstract

**Background:**

Rhinoviruses (RV) are a well-established cause of respiratory illness. RV-C has been associated with more severe illness. We aimed to characterize and compare the clinical presentations and disease severity of different RV type circulating in South Africa.

**Method:**

We performed two analyses of RV-positive specimens identified through surveillance in South Africa across all age groups. First, RV-positive specimens identified through severe acute respiratory illness (SARI) surveillance in four provinces was randomly selected from 2009 to 2010 for molecular characterization. Second, RV-positive specimens identified through SARI, influenza-like illness (ILI) and control surveillance at hospitals and outpatient clinics in during 2012–2013 were used to determine the association of RV type with severe disease. Selected specimens were sequenced, and phylogenetic analysis was performed.

**Results:**

Among the 599 sequenced specimens from 2009 to 2010 and 2012 to 2013, RV-A (285, 48%) and RV-C (247, 41%) were more commonly identified than RV-B (67, 11%), with no seasonality and a high genetic diversity. A higher prevalence of RV infection was identified in cases with SARI [515/962 (26%); aRRR = 1·6; 95% CI 1·21; 2·2] and ILI [356/962 (28%); aRRR = 1·9; 95% CI 1·37; 2·6] compared with asymptomatic controls (91/962, 22%). There was no difference in disease severity between the different type when comparing SARI, ILI and controls.

**Conclusion:**

All three type of RV were identified in South Africa, although RV-A and RV-C were more common than RV-B. RV was associated with symptomatic respiratory illness; however, there was no association between RV type and disease severity.

## Introduction

Pneumonia is a major cause of morbidity and mortality in children worldwide and causes 18% of all deaths in children <5 years of age.^1^ Currently, more than 100 different serotypes of Rhinovirus and three genetically characterized types (RV-A, RV-B and RV-C) have been described.^2^ Although the majority of RV infections are associated with mild disease, their impact on overall morbidity and economic cost worldwide is thought to be considerable.^3^ Some studies have suggested that infection with RV-C may result in more severe illness compared with RV-A and RV-B.^4^

Using real-time PCR methods, we previously reported that RV was identified in 25% of patients that were hospitalized with severe acute respiratory illness (SARI) in South Africa.^5^ RV has also been identified among asymptomatic patients^6^ with a reported prevalence of 12–22% among children and 13% among immune-compromised patients.^7^ RV has a relatively short shedding period in otherwise healthy persons; however, prolonged shedding of over and above 28 days has been reported for immune-compromised patients.^8^ Consequently, the clinical relevance of detecting RV among hospitalized patients is difficult to interpret, especially in a population with a high HIV sero-prevalence. According to statistics of South Africa, 10% of the South African population is HIV-positive. This suggests a high percentage of potentially vulnerable individuals^9^ and highlights the need to determine the association of respiratory viruses like RV with SARI relative to patients with milder illness or without respiratory symptoms.

We investigated the prevalence, epidemiological characteristics, genetic diversity and disease association of RV, including type, among patients with SARI, influenza-like illness (ILI) and asymptomatic controls in South Africa.

## Materials and methods

### Study design and population

#### SARI surveillance

Study samples were obtained from participants enrolled in a prospective hospital-based surveillance programme for SARI initiated in February 2009, which aimed to describe the aetiology and risk factors for acute lower respiratory tract infection (ALRI) in all age groups in South Africa. The methodology and case definitions of this study have been previously described.^5^,^10^ All patients were enrolled only once and followed through until discharged from the hospital.

#### ILI and asymptomatic control surveillance

Study samples were obtained from participants enrolled in an active surveillance programme for ILI, and asymptomatic controls initiated in May 2012 through 2013. Patients presenting with ILI and asymptomatic controls were enrolled at two outpatient clinics serving the population surveyed at two of the SARI sentinel sites: the Gateway Clinic, KwaZulu Natal Province and Jouberton Clinic, Northwest Province. An ILI case was defined as an outpatient of any age presenting with cough duration of ≤7 days with either temperature >38°C or history of fever. ILI cases that were referred for hospitalization subsequent to the visit were not eligible for enrolment.

An asymptomatic control was defined as an individual presenting at the same outpatient clinic with no history of fever, respiratory or gastro-intestinal symptoms during the 14 days preceding the visit. The patients commonly presented to the clinic for visits such as dental procedures, family planning, well baby clinics, voluntary HIV counselling and testing or acute care for non-febrile illnesses. Medical and symptoms history was systematically verified by a trained nurse using a structured checklist. This information was obtained through medical chart review and interview with the patient or legal guardian for children <15 years of age. One HIV-infected and one HIV-uninfected control were enrolled every week in each ILI clinic within each of the following age categories: 0–1, 2–4, 5–14, 15–54 and ≥55 years.

A standardized questionnaire was used to collect demographic and clinical information from each enrolled SARI and ILI case and control. In addition, for SARI cases hospital records were reviewed to assess disease progression and outcome (i.e., discharge, transfer or in-hospital death).

### Sample selection of two groups for molecular characterization

#### 2009–2010 cohort

*SARI cases* were randomly selected from single positive RV SARI patients; specimens were sorted according to randomly assigned numbers, and the first 381 were selected (37%, 381/1039) for molecular characterization.

#### 2012–2013 cohort

*SARI, ILI and controls* for the disease association analysis, we assumed a 25% RV prevalence among cases and a 15% RV prevalence among controls, which resulted in a needed sample size of 214 RV-positive cases in each group to statistically assess significance using a 95% confidence interval and 80% power, and a random selection (as described above) of single RV-positive specimens was characterized further.

### Laboratory testing

#### Rhinovirus detection

Respiratory specimens (i.e., nasopharyngeal aspirates for children <5 years of age and nasopharyngeal and oropharyngeal swabs from individuals ≥5 years of age) were collected, placed in viral transport medium, stored at 4–8°C and transported to the National Institute for Communicable Diseases within 72 hours of collection for testing. All specimens were tested for the presence of 10 respiratory viruses using the real assay as described by Pretorius *et al*.^5^ Among consenting study patients, HIV status was established by enzyme-linked immunosorbent assay (ELISA) or PCR depending on the patients' age.

#### Sequencing of the Rhinovirus VP4/VP2 genomic fragment

A 440 base pair region of VP4 and VP2 was amplified and sequenced for 595 randomly selected RV-positive specimens (single infection) consisting of 381 SARI specimens from 2009 to 2010 and 214 SARI, ILI and control specimens from 2012 to 2013. Briefly, the first round of RT-PCR was performed using primers PR-1 (Forward) and PR-2 (Reverse).^11^ Nested PCR was performed, using primers hrv 01.3^12^ and RV2n with an expected band size of 550 bp.^11^ Amplicons were purified using the ExoSAP-IT enzyme system (USB Corporation, Cleveland, OH, USA) and sequenced using the big dye terminator version 3.1 cycle Sequencing Ready Reaction kit (Life Technologies, Foster City, CA, USA) using nested primers. Sequences were assembled using Sequencher® version 5 (Gene Codes Corporation, MI, USA), and alignments were performed using MAFFT multiple sequence alignment program.^13^ The nucleotide substitution model used in the maximum-likelihood (ML) analysis was determined using jmodeltest^14^,^15^, and the ML trees were generated using phyml 3.0.^15^,^16^

### Statistical analysis

We implemented three multivariable multinomial regression models. First, using the 2009–2010 cohort of SARI cases, we evaluated factors associated with each RV type. For this analysis, the RV-A type was defined as the baseline category as it was the most common type detected. Second, using the 2012–2013 cohort of SARI, ILI and control cases, we evaluated disease severity associated with RV infection comparing the RV prevalence among SARI and ILI cases to controls (reference group). Third, also using the 2012–2013 cohort, we evaluated disease severity associated with RV type by comparing the proportion of RV type among SARI and ILI cases to controls. Statistical significance was defined as *P* < 0·05. The analysis was performed using stata 12 (Stata Corporation, Texas, TX, USA).

#### Ethical considerations

The SARI protocol was reviewed and approved by the University of the Witwatersrand Human Research Ethics Committee (HREC) and the University of KwaZulu Natal Human Biomedical Research Ethics Committee (BREC) protocol number M081042 and BF157/08, respectively. The ILI and asymptomatic controls protocol were reviewed and approved by BREC protocol number (BREC BF 080/12). This surveillance was deemed non-research by the U.S. Centers for Disease Control and Prevention.

## Results

### Phylogenetic comparison of RV strains identified in 2009–2010 and 2012–2013 cohorts

**Figure 1:**
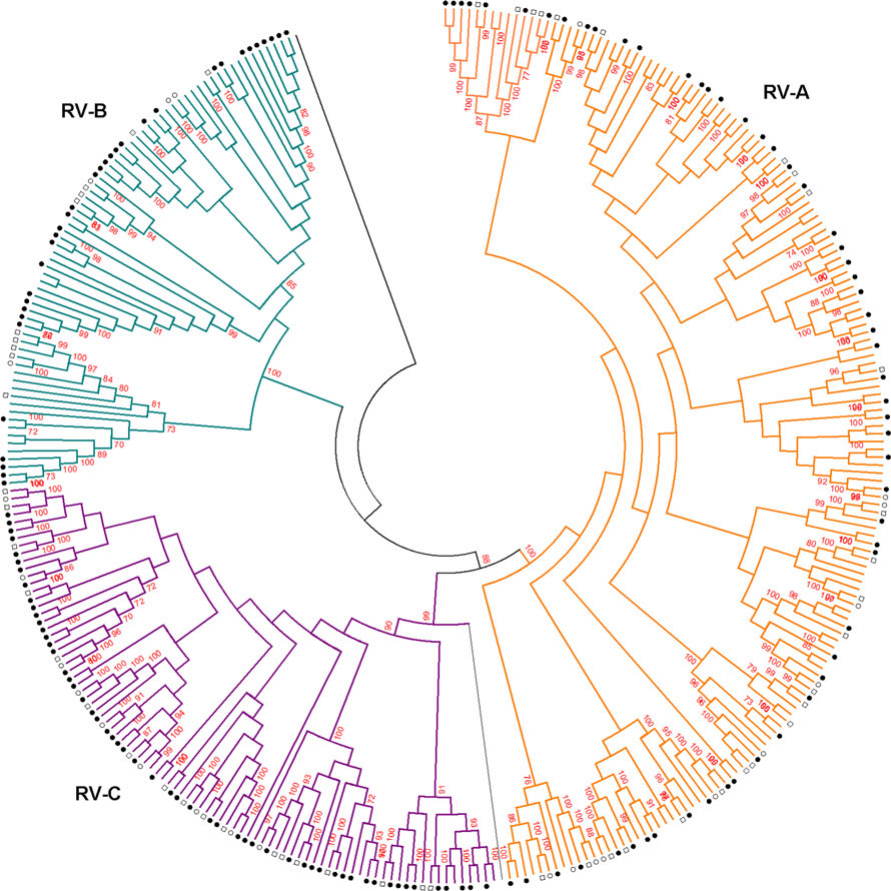
Phylogenetic analysis of RV type by maximum-likelihood method of the VP4/VP2 region, South Africa, 2009–2010 and 2012–2013. Phylogenetic analysis of Rhinovirus sequences from South Africa and reference sequences from Genbank using maximum-likelihood method of the VP4/VP2 region. Sequences with closed circle denotes type identified in SARI patients, those with open squares denotes type identified in ILI patients, while those with open circles denotes type identified in control patients, those without denotation are the reference sequences. Bootstrap values (100 replicates) shown on the branches, with values <70% omitted from the tree.

Maximum-likelihood phylogenetic comparison of clinical specimens from 2009 to 2010 and 2012 to 2013 to international reference sequences indicated that RV-A (285,48%) and RV-C (247,41%) were more commonly identified than RV-B (67,11%) and that the South African sequences for each of the type formed numerous subclusters within each type, with statistically significant bootstrap support (Figure1). Several distinct bootstrap supported clusters of South African viruses were identified in type C (Figure1).

### Epidemiology of RV infection and factors associated with RV type in 2009–2010 cohort

**Figure 2:**
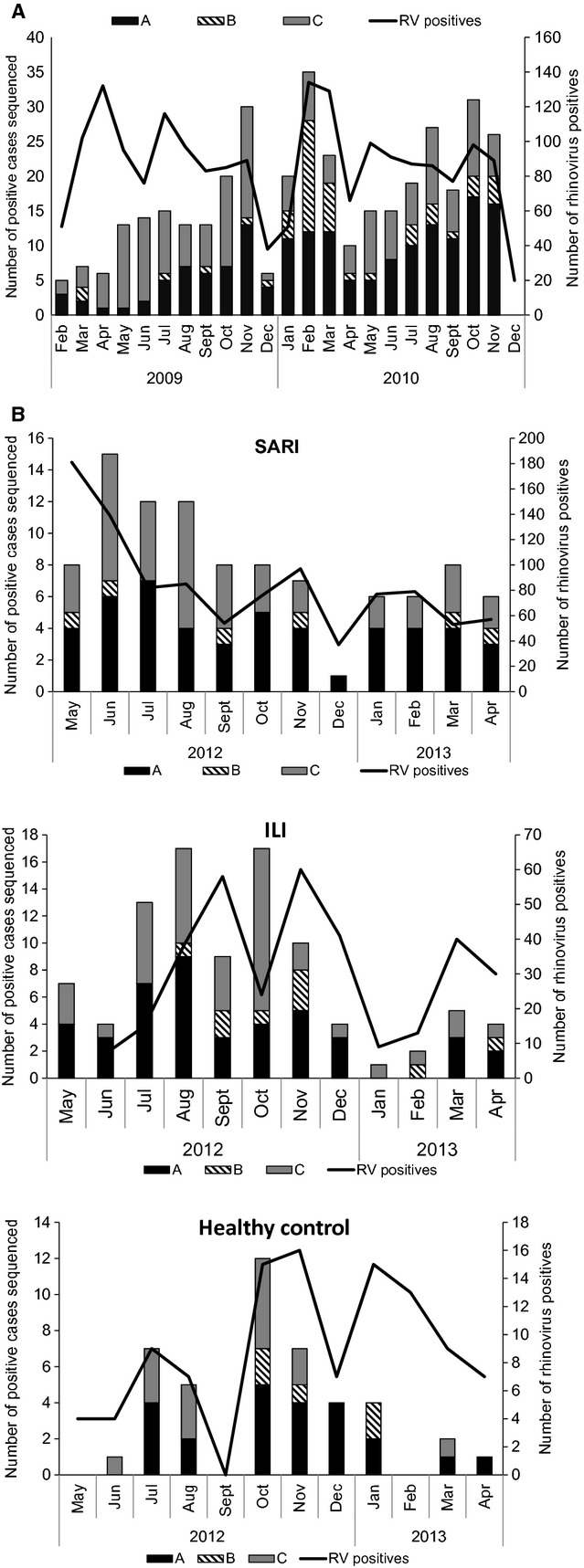
Number of positive cases and detection rate of Rhinovirus by month in South Africa, 2009–2010 SARI (A) and 2012–2013 SARI, ILI and control cases (B).

The 2009–2010 study cohort data were used for this analysis. From February 2009 through December 2010, we obtained laboratory results from 7641 SARI patients. Of these 3171 (41%) were negative for the viral pathogens in our assay. RV was detected in 1949 (25%) subjects, of which it was the only virus identified in 1039 (53%) cases. In multivariable analysis adjusting for age and year of circulation infection, RV-C compared with RV-A type was associated with asthma or having a history of asthma [adjusted relative risk ratio (aRRR) = 3·4 95% CI 1·1; 11·1; Table1]. No difference between RV-B and RV-A type were detected in the multivariable analysis. RV was detected throughout the year with no evident seasonality. RV-A and RV-C cocirculated in 2009–2010, while RV-B was detected sporadically mainly in 2010 (Figure2A).

**Table 1:** Factors associated with RV type among patients hospitalized with severe acute respiratory illness, South Africa, 2009–2010

Factor	Univariable analysis					Multivariable analysis*	
	
	RV-A**	RV-B		RV-C		RV-B	RV-C
				
	*n*/*N* (%)	*n*/*N* (%)	RRR*** (95% CI)	*n*/*N* (%)	RRR*** (95% CI)	RRR† (95% CI)	RRR† (95% CI)
Age group, yrs							
<5	78/162 (48)	9/39 (23)	1	90/156 (58)	1	1	1
5–14	9/162 (6)	1/39 (3)	0·9 (0·1–8·5)	10/156 (6)	0·9 (0·4–2·5)	1·1 (0·1–9·3)	0·9 (0·3–2·2)
15–24	9/162 (6)	2/39 (5)	1·9 (0·3–10·3)	6/156 (4)	0·6 (0·2–1·7)	1·8 (0·3–9·5)	0·6 (0·2–1·9)
25–44	42/162 (26)	19/39 (49)	**3·9 (1·6–9·4)**	38/156 (24)	0·8 (0·5–1·3)	**3·5 (1·4–8·5)**	0·9 (0·5–1·6)
45+	24/162 (15)	8/39 (20)	**2·9 (1·1–8·3)**	12/156 (8)	**0·4 (0·2–0·9)**	2·6 (0·9–7·8)	**0·4 (0·2–0·9)**
Sex (male)	82/162 (51)	13/39 (33)	0·5 (0·2–1·1)	75/156 (48)	0·9 (0·6–1·4)		
Year							
2009	52/162 (33)	6/39 (15)	1	85/156 (54)	1	1	
2010	109/162 (66)	33/39 (85)	**2·7 (1·1–6·7)**	71/156 (46)	**0·4 (0·2–0·6)**	2·0 (0·7–5·2)	**0·4 (0·3–0·7)**
Duration of symptoms >2 days	115/162 (71)	33/39 (85)	2·4 (0·9–5·7)	101/156 (65)	0·7 (0·5–1·2)		
Length of hospitalization >5 days	68/161 (42)	25/39 (64)	**2·4 (1·2–5·0)**	58/155 (37)	0·8 (0·5–1·3)		
HIV infection	71/153 (46)	26/37 (70)	**2·7 (1·2–5·9)**	60/135 (44)	0·8 (0·5–1·3)		
Asthma††	5/162 (3)	1/39 (2)	0·8 (0·1–7·2)	10/156 (6)	2·1 (0·7–6·4)	0·6 (0·1–5·5)	**3·4 (1·1–11·1)**
Underlying illness†††	15/162 (9)	2/39 (5)	0·5 (0·1–2·4)	16/156 (10)	1·1 (0·5–2·3)		
Oxygen therapy	56/161 (35)	20/39 (51)	2·0 (0·9–4·0)	63/156 (40)	1·3 (0·8–2·0)		
Patient died	8/162 (5)	1/39 (3)	0·5 (0·1–4·2)	8/156 (5)	1·0 (0·4–2·8		

* Reference group for the multinomial regression model.

** Unadjusted relative risk ratio (RRR) at univariable analysis.

*** Adjusted relative risk ratio (aRRR) at multivariable analysis.

† Only covariates significant at the multivariable analysis are reported.

†† Asthma was defined in our database as a history of asthma no distinction was made if they were undergoing an exacerbation of their asthma.

††† Underlying illness includes: chronic lung diseases, cirrhosis/liver failure, chronic renal failure, heart failure, valvular hearth disease, coronary heart disease, immunosuppressive therapy, splenectomy, diabetes, burns, kwashiorkor/marasmus, nephritic syndrome, spinal cord injury, seizure disorder or emphysema. RRR highlighted in bold indicates factors significant at *P* < 0·05.

### Association of RV infection and RV type with respiratory disease severity in 2012–2013 cohort

The 2012–2013 study cohort data were used for this analysis. From May 2012 through April 2013, we obtained laboratory results from 3907 patients, of which 2125 (54%) had SARI, 1325 (23%) had ILI and 457 (11%) were controls. Children <5 years of age accounted for 35% (743), 23% (299) and 26% (119) of the SARI, ILI and controls, respectively (*P* = 0·226). RV was identified in 24% (515/2125), 27% (356/1325) and 20% (91/457) of SARI, ILI and controls, respectively. On multivariable analysis adjusting for age (<5, 5–14, 15–44 and ≥45 years age groups; *P* = 0·003) and HIV status (*P* = 0·012), RV infection was associated with both ILI (aRRR: 1·9; 95% CI 1·4–2·6) and SARI cases (aRRR: 1·6; 95% CI 1·2–2·2) compared with controls.

No significant difference was observed between the different RV type and disease severity among the characterized cases (results not shown). RV was detected with no evident seasonality. RV-A and RV-C cocirculated in 2012–2013, while RV-B was detected sporadically throughout (Figure2B).

## Discussion

We describe the RV type circulating among patients from all age groups with acute upper and lower respiratory tract infections and controls in South Africa. While we detected a statistically significant difference in the prevalence of RV among SARI and ILI cases compared with controls, the elevated positivity rate of RV among controls indicates that RV may act as pathogen but could also be present in asymptomatic infections. This suggests that only a proportion of RV infections (along with other factors such as viral load and host interactions) may be responsible for the clinical disease that manifests as ILI or SARI.

We did not identify any difference in disease severity due to different RV type. Similar results have been reported by a study in Thailand^17^; whereby a RV prevalence of 19% in outpatients with ILI and 9% in controls was observed, and no difference in disease severity by RV type was identified. The study in Thailand^17^ and a more recent study conducted in Kenya^18^ showed similar distributions of RV type that we observed in South Africa: RV-A and RV-C cocirculated with no clear seasonality, and RV-B was observed sporadically.

We found that HRV-C is not associated with more severe disease, but does appear to be associated with a history of asthma. This suggests that HRV-C induces asthma, or alternatively, that asthmatic children are vulnerable to HRV-C infections. Studies have shown HRV-C was not only related to wheezing illnesses and asthma, but was also associated with an increased risk of prior and subsequent hospital respiratory admissions.^7^,^19^–^22^

Our study has several limitations. We only recorded in the initial interview if participants had a history of asthma. We did not follow up on participants over the course of the study to determine whether the RV infection led to a diagnosis of wheezing or asthma or whether any viral nucleic acids detected using RT-PCR may represent the pre-syndromic phase of a viral infection. Also, the case definition for SARI and ILI was restricted to patients with duration of symptoms ≤7 days, so we could have missed some cases that might have more prolonged illness. The collection of different types of samples may potentially affect the sensitivity of RV detection. However, when comparing the detection rate of respiratory viruses in different samples, Blaschke *et al*.^23^ have shown that non-invasive methods for collecting respiratory samples can be used to identify respiratory viruses with multiplex PCR testing. Finally, while adjusting for age and HIV status in our analyses on association with disease severity, we were not powered to implement age- and HIV-stratified analysis, hindering the ability to detect differences among different groups.

In conclusion, we showed that there was a high diversity in the sequences of the RV type that circulated in South Africa. RV is detected in a proportion of outpatient and hospitalized respiratory disease but is also detected in individuals with no history within the past 14 days of respiratory illness, which suggests that rhinovirus can act as a disease causing agent and be found in asymptomatic infection. Further studies are necessary to determine if other factors such as viral load or host interactions play a role in RV-associated disease.
